# PLAS-5k: Dataset of Protein-Ligand Affinities from Molecular Dynamics for Machine Learning Applications

**DOI:** 10.1038/s41597-022-01631-9

**Published:** 2022-09-07

**Authors:** Divya B. Korlepara, C. S. Vasavi, Shruti Jeurkar, Pradeep Kumar Pal, Subhajit Roy, Sarvesh Mehta, Shubham Sharma, Vishal Kumar, Charuvaka Muvva, Bhuvanesh Sridharan, Akshit Garg, Rohit Modee, Agastya P. Bhati, Divya Nayar, U. Deva Priyakumar

**Affiliations:** 1 Centre for Computational Natural Sciences and Bioinformatics, International Institute of Information Technology, Hyderabad, 500032 India; 2 UM-DAE-Centre For Excellence In Basic Sciences, University of Mumbai, Vidyanagari, Mumbai, India; 3 Centre for Computational Science, Department of Chemistry, University College London, London, WC1H 0AJ United Kingdom; 4 Department of Materials Science and Engineering, Indian Institute of Technology Delhi, Hauz Khas, New Delhi, 110016 India

**Keywords:** Computational chemistry, Cheminformatics

## Abstract

Computational methods and recently modern machine learning methods have played a key role in structure-based drug design. Though several benchmarking datasets are available for machine learning applications in virtual screening, accurate prediction of binding affinity for a protein-ligand complex remains a major challenge. New datasets that allow for the development of models for predicting binding affinities better than the state-of-the-art scoring functions are important. For the first time, we have developed a dataset, PLAS-5k comprised of 5000 protein-ligand complexes chosen from PDB database. The dataset consists of binding affinities along with energy components like electrostatic, van der Waals, polar and non-polar solvation energy calculated from molecular dynamics simulations using MMPBSA (Molecular Mechanics Poisson-Boltzmann Surface Area) method. The calculated binding affinities outperformed docking scores and showed a good correlation with the available experimental values. The availability of energy components may enable optimization of desired components during machine learning-based drug design. Further, OnionNet model has been retrained on PLAS-5k dataset and is provided as a baseline for the prediction of binding affinities.

## Abstract (summary)

**Table:** 

Measurement(s)	Binding Affinity
Technology Type(s)	Molecular dynamics simulation/MM-PBSA
Factor Type(s)	3D-protein structures
Sample Characteristic - Organism	NA
Sample Characteristic - Environment	NA
Sample Characteristic - Location	NA

## Background & Summary

The task of predicting binding affinity of a protein-ligand (PL) complex is of cardinal significance in the drug design pipeline^1^. In general, determining the binding affinities of PL complex through experimental assays is laborious and economically non-viable. To mitigate the investments in drug discovery, in-silico methods have been adopted over traditional experiments in initial stages of drug design. Experimentally inaccessible molecular interactions and mechanisms can be studied through computational methods. Computer-aided drug design (CADD) is one such promising area of drug discovery and helps to predict the best interaction model between a PL and use scoring functions to estimate the strength of the binding. In recent decades, researchers have increasingly recognized that molecular dynamics simulation (MD) helps to overcome the major limitations of docking calculations that do not sample protein conformational rearrangements during the ligand-binding process. MD simulations based on binding affinity calculations using molecular mechanics with Poisson-Boltzmann (MM-PBSA/MM-GBSA) are therefore expected to provide significant contributions to real-world problems such as identification of hit and lead optimization. The most important post-processing methods for calculating the binding free energy of a PL complex include molecular mechanics with Poisson-Boltzmann/Generalized-Born and surface area (MM-PBSA/MM-GBSA), and alchemical approaches like thermodynamic integration and free-energy perturbation (FEP)^2^. Apart from these methods, machine learning (ML) models have also been used for binding affinity predictions (BAP)^3^. ML models can enhance data-driven decision-making and have the potential to speed up the drug discovery process. The current ML models developed for BAP are grouped by the different types of encoding, topology, and atom pairs.

Interaction fingerprints framework used for binding site comparison has proven to be successful in many applications, ranging from assessment of docking poses to the evaluation of novel PL complexes^4^. Some of the applications include structural Protein-Ligand interaction fingerprint^5^, Protein-ligand extended connectivity fingerprint^6^ and most recently Substructural Molecular and Protein-Ligand Interaction Pattern Score^7^. In 3D grid-based studies, PL complex is represented using a 3D grid representation. AtomNet was one of the first published models that used a convolutional neural network for affinity prediction^8^. Few other models include KDEEP^9^, Pafnucy^10^, DeepAtom^11^, and BindScope^12^.

Another deep learning method that could reach the state-of-the-art performance in predicting PL interaction is graph neural network. Few applications include GraphBAR^13^, structure-aware interactive graph neural network^14^, the model developed by Lim *et al*.^15^, and PotentialNet^16^. Apart from these models, other models such as MathDL^17^ and TopologyNet^18^ encode interactions PL using methods from algebraic topology. Models such as DeepBindRG^19^, DeepVS^20^, and OnionNet^21^ are focused on interacting atom environments of complex structures.

A number of datasets facilitate the development of ML-based scoring functions^22^ for BAP. Such ML scoring functions use PL information either as a complex or as two different entities. Several benchmarking datasets are publicly available. The BindingMOAD^23^, PDBbind^24^, and CSAR datasets^25^ were compiled to aid in the prediction of binding affinities based on experimental PL complex structures. The KIBA^26^ and DAVIS^27^ dataset highlights the bioactivities of the kinase protein family and their relevant inhibitors and does not include the structural information of PL complexes. The DUD and DUD-E datasets^28^ were designed to evaluate docking enrichment performance. However, the existing datasets are limited to crystal structures of PL complex despite the widely accepted role of protein flexibility in molecular recognition^29^. This simplified description of the complex narrows down the accuracy of the binding pose prediction and their corresponding scoring functions^30^. Herein, MD simulations play a major role in capturing the conformational changes in the complex structure thereby helping in the accurate prediction of binding affinity. This could also improve the size of the diverse datasets and enhance the existing scoring functions based on energetic contributions to binding affinities. In existing datasets, energy components are unavailable, although they are highly important for lead optimization and target-specific drug design. MM-PBSA is a method that provides individual energy components along with the overall binding affinities from MD trajectories. In recent years, MM-PBSA has become a popular method to estimate the ligand binding affinities and it has several applications^31^. Few examples include, development of potential anticancer compounds^31,32^, understanding resistance mechanism of drugs^33^, neural disorder^34^, blood disorder^35^, immune disorder^36^, inflammatory disorder^37^, metabolic disorder^38^, and many other major diseases^39,40^. Apart from these PL interactions, MM-PBSA calculations also play a major role in other biomolecular studies such as protein folding, protein-protein interaction^41^, and others^42^. Various studies also highlight the successful applications of MM-PBSA in virtual screening for identification of potential lead compounds^43^. The most recent application includes identification of suitable inhibitors for COVID-19 targets and also repurposing of existing FDA approved drugs^44^.

In this work, we employed MD simulations on 5000 PL complexes to calculate the binding affinities using MM-PBSA approach. To best of our knowledge, this is the first MD-based dataset that provides binding affinities along with non-covalent interaction components. Comparisons have been made by calculating the correlation coefficients between experimentally determined values to that of calculated affinities (MM-PBSA and Docking). As a baseline, we have trained the OnionNet framework on our dataset. We believe that PLAS-5k and further work in this direction will provide the necessary impetus for the development of data-driven methods for drug design tasks such as hit identification, lead optimization, de novo molecular design, etc.

## Methods

### Data curation

In this article, as a first step towards the development of dataset, we have selected 5000 complexes randomly from PDB^23^ based on the following criteria (i) In these complexes, ligand is chosen to be either a small organic molecule or a peptide, (ii) the complex structures within 2.5 Å resolution.

### System preparation

**Fig. 1:**
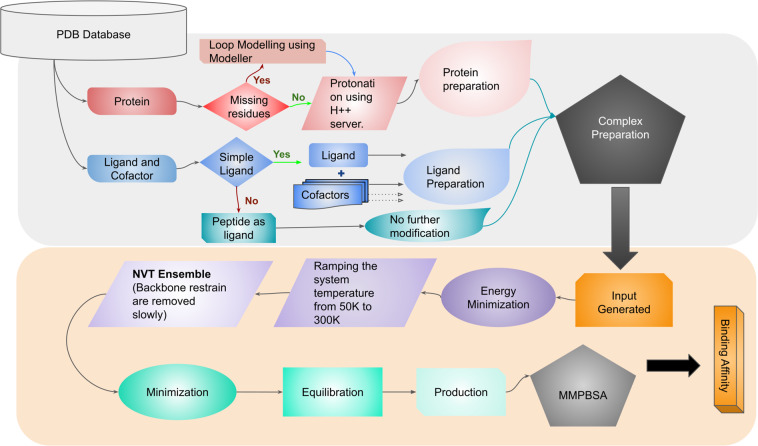
Protocol for input preparation and simulations.

Each protein-ligand complex chosen is composed of protein, ligand, cofactor(s) and crystal water molecules. The procedure of preparing the complexes for MD simulations is discussed in detail in the following sections, and is shown in Fig. 1.

#### Protein preparation

Most of the chosen experimental protein structures are monomers, while few can be functional as multimers. In cases of multimers, the subunits within a distance of 8 Å of ligand molecule were considered for complex preparation. In case of missing residues, MODELLER program was used to build the missing residues in PDB structures as loop regions^45^. Further, protonation states of the residues in the protein structures were determined using the H++ server^46^ at the physiological pH of 7.4. For the simulations, Amber ff14SB parameters were used for proteins^47^.

#### Ligand preparation

The information of total charge on the ligand was retrieved using ligand-expo and hydrogen atoms were added to the ligand using GaussView^48^ in appropriate positions^49^. Similar procedure were adopted for the cofactors. The forcefield parameters for ligand and cofactors were obtained from General AMBER force field (GAFF2)^50^ using Antechamber program^51^ of Ambertools^52,53^. AM1-BCC charges were assigned to the atoms of ligand and cofactor(s). In case of peptides, Amber ff14SB^47^ forcefield was used.

#### Complex preparation

As water molecules play an important role in mediating protein-ligand interactions, the crystal waters associated with the selected subunits of proteins have been considered for the studies.The “tleap” program of AmberTools^52,53^ was used to generate a complex. The systems were solvated in an orthorhombic water box with a 10 Å extension from the protein. To maintain the charge neutrality of the system, counter ions (Na^+^ or Cl^−^) were added.

### Simulation setup

#### Energy minimization

Minimization was performed in two steps. First, the protein backbone atoms were restrained using a harmonic potential with a force constant of 10 kcal/mol/Å^2^ in 1000 step minimization using L-BFGS minimizer was carried out. Further, the spring constant was reduced in ten steps and energy minimization was performed. In each step, the force constant was scaled by half. Finally, the harmonic restraints were turned off and minimization was carried out for another 1000 steps.

#### Simulating to target temperature (300 K)

After energy minimization, short MD simulation was performed with a timestep of 2 fs in NPT ensemble, with position restraints on backbone atoms using harmonic potential with spring constant of 1 kcal/mol/Å^2^. The particle mesh Ewald (PME) method was used to compute the long range interactions and the non-bonded interactions were truncated at 10.0 Å. The bonds involving hydrogen atoms were constrained. The temperature of the system was maintained using Langevin thermostat with a friction coefficient of 5 ps^−1^. The system temperature was raised from 50 K to 300 K by increasing the temperature by 1 K in every 100 steps (200 fs). Finally, after reaching target temperature (300 K), simulations were performed for 1 ns in the NVT ensemble.

#### Multiple independent simulations

Studies have reported that many short run independent simulations are more effective than a single long run, and it will decrease the uncertainty for the predicted binding affinities^54–56^. In general, the independent simulations are performed with different set of random initial velocities and initial structures taken during the minimization. The initial structures were generated from energy minimization in 40000 steps. At every 10000 steps, the structures were saved to start five independent simulations (including the starting structure).

In the next stage, all the restraints were released and the atoms were allowed to move freely. The system was equlibrated in the NPT ensemble at 300 K and 1 atm using a Langevin thermostat and Monte Carlo barostat for 2 ns. Finally, a production run was performed for 4 ns in the NPT ensemble, and the trajectories were saved every 100 ps for the post-processing analysis and free energy calculations. Molecular dynamics simulations have been carried out using the OpenMM 7.2.0 program^57^.

### Molecular-Mechanics Poisson Boltzmann Surface Area (MM-PBSA) calculations

MM-PBSA has been extensively used in CADD, as it is less expensive compared to alchemical free energy methods. Binding free energy of a PL complex is calculated according to the following equation.

1 $$\Delta {G}_{MM-PBSA}=\Delta {E}_{MM}+\Delta {G}_{Sol}$$

Further, Δ*E*_*MM*_ is divided into sum of electrostatic interaction energy Δ*E*_*ele*_, and van der Waals interaction energy Δ*E*_*vdw*_ (Eq. (2)). The solvation free energy Δ*G*_*sol*_, is defined as sum of polar Δ*G*_*pol*_, and non-polar contributions Δ*G*_*np*_ (Eq. (3)).

2 $$\Delta {E}_{MM}=\Delta {E}_{ele}+\Delta {E}_{vdw}$$

3 $$\Delta {G}_{Sol}=\Delta {G}_{pol}+\Delta {G}_{np}$$

Polar solvation energy, Δ*G*_*pol*_ was calculated using the PBSA method as implemented in the AMBER20 program and non-polar contributions were determined using Linear Combinations of Pairwise Overlap (LCPO) method^58^.

Both experimental and CADD have highlighted the role of water molecules in PL binding as they aid in water mediated hydrogen bond interactions^59–61^. In our study we have considered two water molecules (see SI for more details and Supplementary Figure S1), which are near to the PL interaction site. The internal dielectric constant 4 was considered, as several studies reported good performance in predicting binding affinity^62–64^. The binding affinity for each complex was calculated by single trajectory approach. From the complex, protein and ligand are extracted and their affinities were calculated separately. The reported binding affinities are the mean of the Δ*G* calculated from all the five independent runs.

### Docking protocol

In structure-based drug design, docking studies have been used to determine the binding pose and affinities. The docking results are obtained by the simplified description of the complex which lacks true dynamics of the system and explicit water molecules^30^. On the other hand, it is been reported that end-point methods, such as MM-PBSA/MM-GBSA, are based on snapshots of MD simulations trajectories and they tend to overcome the limitations of docking and provide more accurate results than docking scoring functions. In this work, docking studies were performed for structures with known experimental binding affinities using AutoDock vina^65^. The crystal structures of all PL complexes were retrieved from PDB database and were refined by removing heteroatoms. Further, hydrogen atoms were added and Kollman charges were assigned to the protein structures. For ligands, Gasteiger partial atomic charges were assigned and all flexible torsion angles were defined using AUTOTORS. The active site of each target was discretized through a grid and the docking calculations were performed with default parameters^66^.

## Data Records

PLAS-5k dataset (https://hai.iiit.ac.in/datasets.html) can be searched using the PDB id as a query and an example of data retrieval from the PLAS-5k database is illustrated in Supplementary Figure S2. After submitting the query the results are displayed and it gives information on the total binding affinity and different energy components like van der Waals interaction energy, electrostatic energy, polar and non-polar solvation energies. Structural visualization of the protein-ligand complex is available for each entry. The initial structures of all the 5000 protein ligand complexes are available in PDB format and the csv file containing information about binding affinity components can be accessed through figshare^67^.

## Technical Validation

### Overall structures of the protein-ligand complexes

In the present work, we performed MD simulations to capture several conformations of the PL complex to incorporate the flexibility of protein in binding affinity calculations. The experimental structure of a complex is taken as a reference in the RMSD calculation of both protein and ligand over the simulation trajectory. In order to capture the conformations of ligand, the structure of the protein was superimposed primarily and the RMSD of protein and ligand was measured separately for all five independent runs. The cumulative RMSD of protein and ligand for each of the complexes is calculated over all 200 frames (40 from each simulation), and the corresponding distributions are shown in Supplementary Figure S3. The long tail in the distributions are due to the presence of flexible groups present in protein (loops) and ligand. Since the RMSD for ligands peak at <1 Å and the majority fall below 3 Å, the ligands remain stably bound throughout the simulations. Our dataset covers wide range of ligands and the distribution of molecular weights of these ligands is shown in Supplementary Figure S4.

Experimentally, the binding affinity of a protein-ligand complex is expressed in terms of dissociation constant (K_*d*_) or inhibition constant (K_*i*_). This experimentally determined binding equilibrium constant is related to the binding free energy as,

4 $$\Delta {G}_{expt}=-{k}_{B}T\;ln{K}_{i}=-{k}_{B}T\;ln(1/{K}_{d})$$

MM-PBSA approach has been widely accepted as an efficient and reliable free energy method in estimating PL binding interactions and has high correlation with experimental binding affinity^68^ especially for a given protein with respect to multiple ligands. A combination of interaction energetic components from MM-PBSA and ML methods help in developing models that could identify suitable inhibitors for a specific target^69^. The calculated binding free energies using MM-PBSA method span a wide range of values capturing a broad distribution suitable for developing ML models (Supplementary Figure S5). Having a knowledge on these large interval values of calculated binding affinity for diverse dataset, would help in extracting feature representation of PL complexes and train reliable regression models that can help in predicting binding affinity of a novel complex, and for use in other applications such as molecule generation.

### Comparison of experimental *vs* calculated binding affinities

**Fig. 2:**
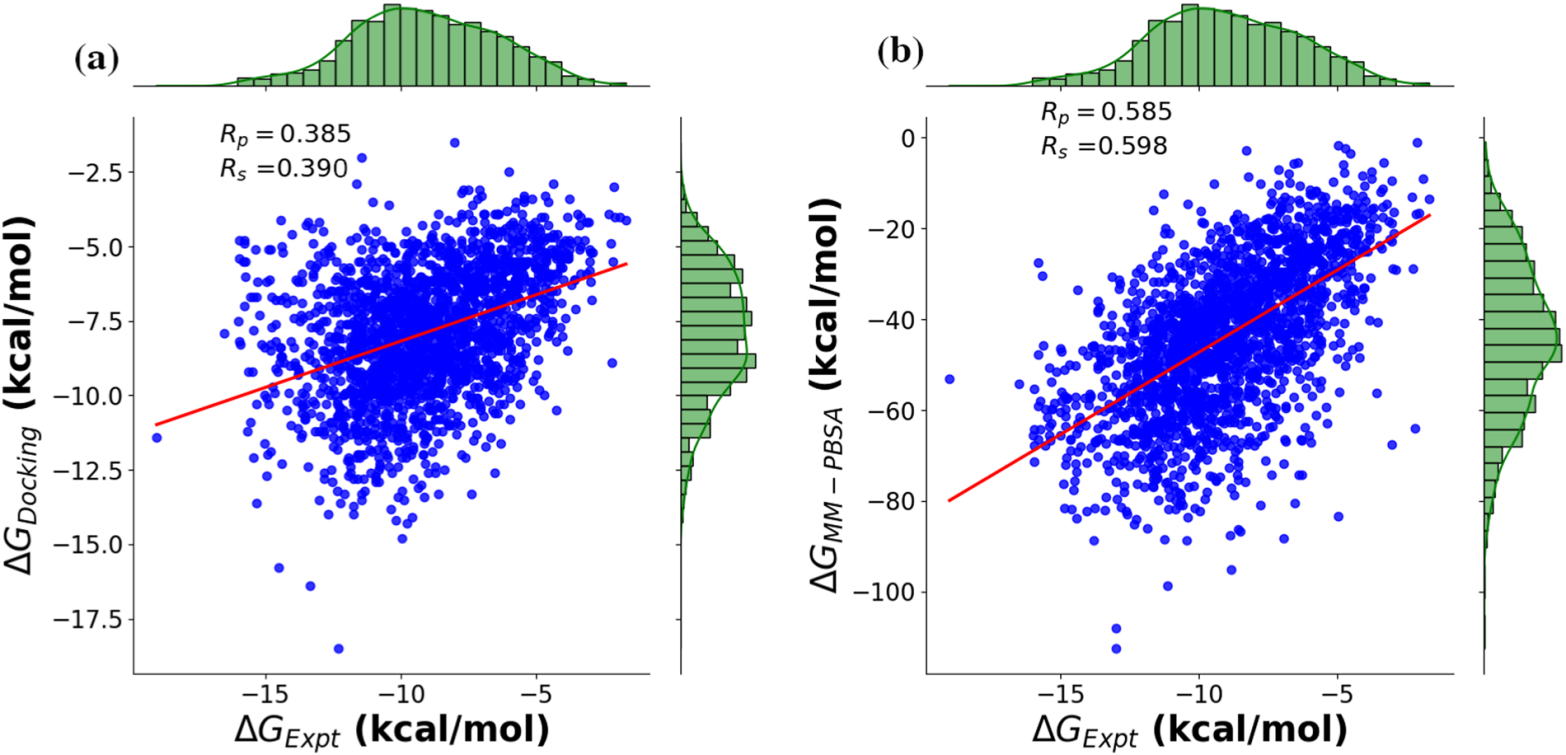
Correlation plots between the experimental and calculated binding affinities for a subset with 2000 pdbids. The binding affinities are calculated (**a**) using Auto-dock Vina, and (**b**) using MM-PBSA.

For comparison study, we made a subset (2000 complexes) of 5000 complexes, whose experimental binding affinities are known. The calculated binding affinities based on docking studies and MM-PBSA method were compared with the experimental values. The Spearman rank correlation coefficient (R_*s*_) and Pearson correlation coefficient (R_*p*_) were used to evaluate the ranking of binding affinities and their correlation with experimental data respectively. As seen in Fig. 2, the (R_*p*_) was 0.385 for docking studies with (R_*s*_) of 0.390, while the studies based on MM-PBSA show relatively stronger correlation with (R_*p*_) and (R_*s*_) of 0.585 and 0.598 respectively. This indicates that ML based scoring functions developed using PLAS-5k dataset are expected to be more reliable than the traditional scoring functions.

### Class specific performance

The dataset was classified into seven different classes as follows: (i) Transferases, (ii) Hydrolases, (iii) Isomerases, (iv) Oxido-reductases, (v) Ligases, (vi) Lyases, and (vii) Others. These enzymes are essential biological catalysts involved in a number of chemical transformations pertaining to life. From the Table 1 and Supplementary Figures S6, S7 it can be noted that the binding affinities predicted through MM-PBSA shows good correlation with the experimental value for most of the classes compared to docking affinities.

**Table 1:** Correlation between experimental and predicted binding free energies for different enzyme classes on a subset of PLAS-5k containing 2000 pdbids, whose experimental binding affinities are available.

Enzyme class	Number of complexes in each class	${{\bf{R}}}_{{\boldsymbol{p}}}^{{\boldsymbol{Docking}}}$	${{\bf{R}}}_{{\boldsymbol{s}}}^{{\boldsymbol{Docking}}}$	${{\bf{R}}}_{{\boldsymbol{p}}}^{{\boldsymbol{MM-PBSA}}}$	${R}_{{\boldsymbol{s}}}^{{\boldsymbol{MM-PBSA}}}$
Transferase	613	0.456	0.454	0.521	0.517
Hydrolase	572	0.345	0.357	0.620	0.670
Oxido-reductases	273	0.475	0.413	0.325	0.328
Isomerase	56	0.603	0.625	0.694	0.707
Ligase	72	0.432	0.419	0.667	0.662
Lyase	36	0.438	0.358	0.534	0.492
Others	378	0.411	0.403	0.529	0.552

In this subset peptide inhibitors were not considered.

### Target-specific performance: experimental *vs* docking and MM-PBSA

#### Performance of HIV-1 protease targets

HIV-1 Protease is an essential enzyme in the life cycle of HIV as they play an important role in viral replication and maturation. The discovery of HIV-1 protease inhibitors in the last 25 years is a major success in structure based drug design. There are totally nine FDA approved protease inhibitors. A lot of efforts have been made in drug discovery process in development of next-generation protease inhibitors beyond the currently approved protease inhibitors. This shows that until today, HIV-1 protease continues to be one of the attractive targets as they continue to play an important role in drug discovery^70–74^.

**Fig. 3:**
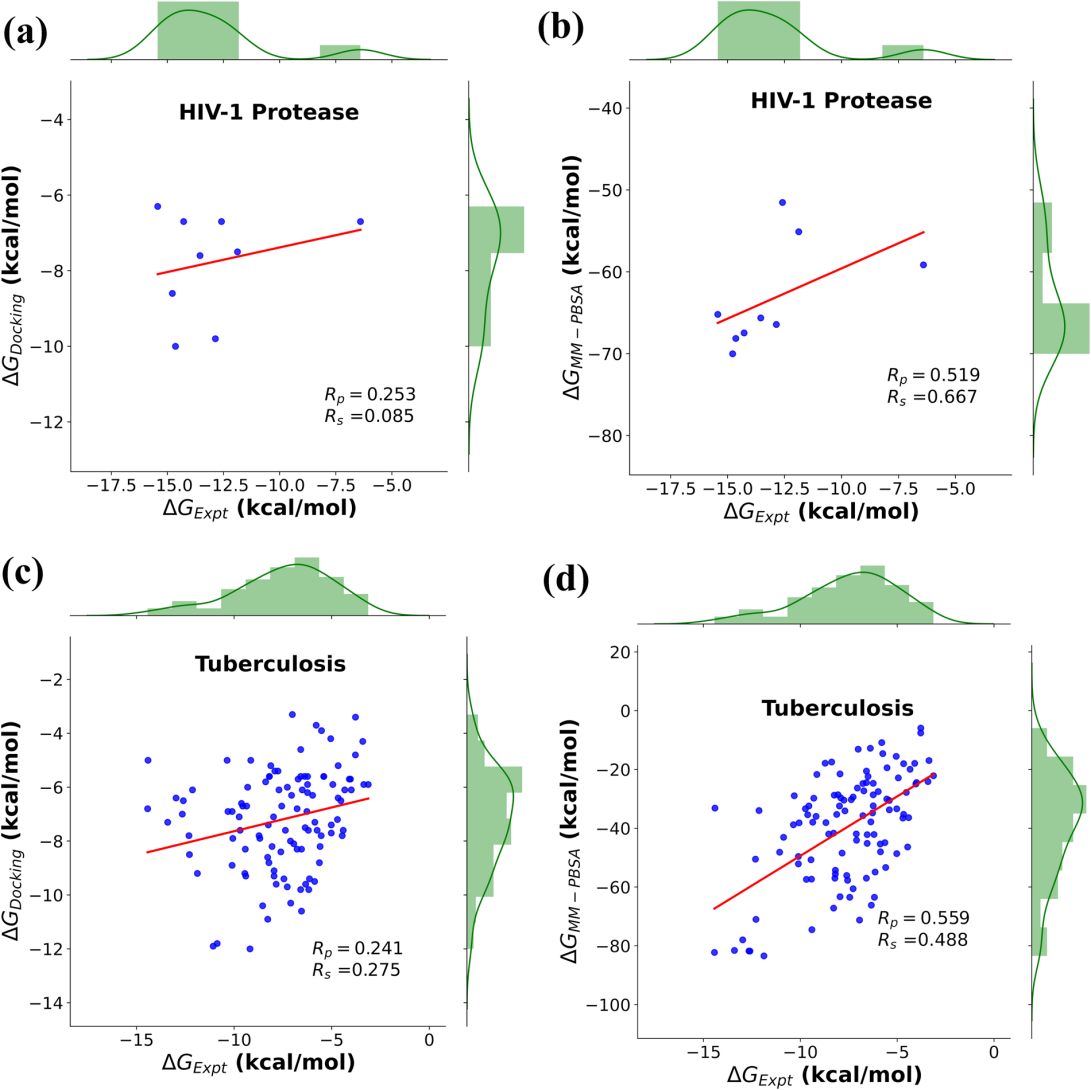
Prediction of binding affinity based on correlation with experimental data: FDA approved drugs for HIV-I protease targets (**a**) Experimental vs Docking, (**b**) Experimental vs MM-PBSA; For Tuberculosis targets - (**c**) Experimental vs Docking (**d**) Experimental vs MM-PBSA.

Docking studies of HIV-1 protease with FDA approved drugs shows that (R_*p*_) and (R_*s*_) were 0.25 and 0.09 respectively (Fig. 3a). As shown in Fig. 3b, in case of MM-PBSA calculations, the simulation results show good correlation of 0.52 (R_*p*_) and 0.68 (R_*s*_). The linear correlation coefficient (R_*p*_) is marginally good, but the Spearman ranking coefficient showed better performance than that of R_*p*_, which is more essential characteristic in drug discovery.

#### Performance of tuberculosis targets

Tuberculosis (TB), a contagious and potentially fatal disease continues to be a major health problem worldwide. Though tremendous progress has been made in anti-TB therapy over the last seven decades to eradicate the disease, TB continues to affect millions of people worldwide^75^. Numerous efforts have been made in drug discovery to search new antitubercular agents that can inhibit the drug resistant strains^76,77^. With this motivation, we selected TB targets to assess the performance of our dataset. As observed for HIV-1 protease, even the TB targets showed better performance in case of MM-PBSA calculations with correlation values (R_*p*_) and (R_*s*_) ranking of 0.56 and 0.49 respectively, whereas the docking results showed values of 0.24 (R_*p*_) and 0.28 (R_*s*_). The correlation plots for tuberculosis targets are shown in Fig. 3(c,d).

#### Components of the binding free energies

Non-bonded/non-covalent interactions play a crucial role in stabilizing the protein-ligand complexes and a detailed understanding of these interactions can provide valuable insights in drug design. One of the advantages about PLAS-5k is that it provides protein-ligand interactions in terms of electrostatic interactions, van der Waals interactions, polar and non-polar contributions to solvation free energy. The distribution plots are shown in Supplementary Figure S8. A knowledge of these individual energy components (Eq. (1)) could help the researchers to have an tailored procedure in lead optimization of drug discovery process.

### Machine learning benchmark

**Fig. 4:**
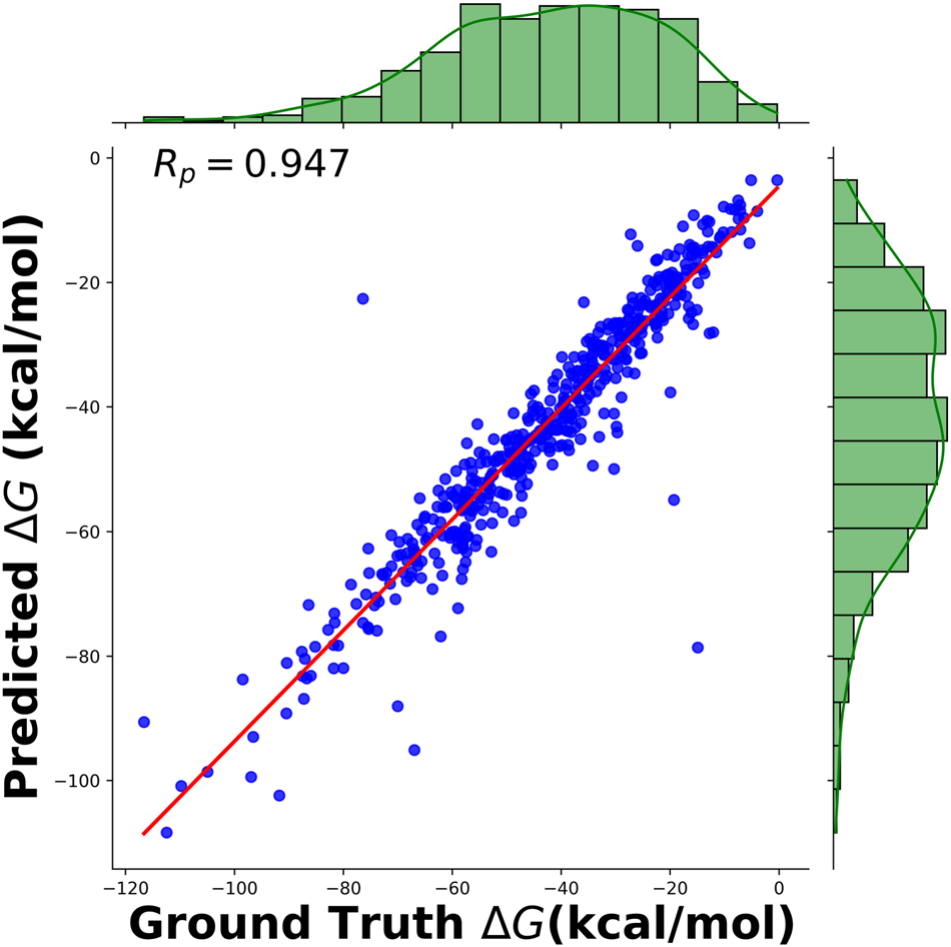
Pearson correlation coefficient after training OnionNet on PLAS-5k database.

Prediction of binding affinity of a PL complex is a critical step in drug design, and ML methods have begin to make significant contributions. One of the pioneering model is OnionNet^21^. Taking various features derived from 3D molecular structure as a input and known binding affinities it predicts binding affinity for a unknown complex via use of Convolutional Neural Network (CNN). PLAS-5k data, was trained and tested using OnionNet model. A 10-fold validation was employed, where the dataset was divided into 10 equal parts and 9-parts were used for training the model, rest for testing. This was employed due to the size constraint of the dataset. The average RMSE across all the 10-fold split was 5.7 kcal/mol and with an R_*p*_ of 0.96, as shown in Fig. 4.

## Supplementary information

Supplementary Information for: PLAS-5k: Dataset of Protein-Ligand Affinities from Molecular Dynamics for Machine Learning Applications

## Data Availability

No custom code was used in the creation of this database. We used OnionNet^21^ http://github.com/zhenglz/onionnet/ ML model to train on PLAS-5k dataset. Ambertools^52^, GaussView^48^, MODELLER^45^, and H++ server^46^ were used for preparation of complex containing protein, ligand, and cofactor(s). MD simulations were carried using OpenMM 7.2.0 program^57^.
