# Supplementary material for: PLAS-5k: Dataset of Protein-Ligand Affinities from Molecular Dynamics for Machine Learning Applications
Source: Sci Data. 2022 Sep 7;9:548. doi: 10.1038/s41597-022-01631-9 (PMC9451116; doi:10.1038/s41597-022-01631-9)
Supplement: Supplementary file 1 — Supplementary Information for: PLAS-5k: Dataset of Protein-Ligand Affinities from Molecular Dynamics for Machine Learning Applications [file 41597_2022_1631_MOESM1_ESM.pdf]

Supplementary Information for: PLAS-5k: Dataset of Protein-Ligand Affinities from Molecular Dynamics for Machine Learning Applications

Divya B. Korlepara[1], C. S. Vasavi[1], Shruti Jeurkar[1], Pradeep Kumar Pal[1], Subhajit Roy[1,2], Sarvesh Mehta[1], Shubham Sharma[1], Vishal Kumar[1], Charuvaka Muvva[1], Bhuvanesh Sridharan[1], Akshit Garg[1] , Rohit Modee[1], Agastya P. Bhati[3], Divya Nayar[4,*], and U. Deva Priyakumar[1,*]

[1]*Centre for Computational Natural Sciences and Bioinformatics, International Institute of Information Technology, Hyderabad, 50032, India.*

[2]*UM-DAE-Centre For Excellence In Basic Sciences, University of Mumbai, Vidyanagari, Mumbai, India.*

[3]*Centre for Computational Sciences, Department of Chemistry, University College London, London WC1H 0AJ, United Kingdom.*

[4]*Department of Materials Science and Engineering, Indian Institute of Technology Delhi, Hauz Khas, New Delhi, 110016, India.*

E-mail: divyanayar@iitd.ac.in,deva@iiit.ac.in

Contents

Fig S1: Change in Pearson correlation with the inclusion of explicit water molecules near protein as well as ligand at binding site in the MMPBSA calculations. This analysis performed for 75 Protein-ligand complexes which are part of 2000 PL complex set used for comparsion study. **3**

Fig S2: Screenshot of the PDB Viewer **4**

Fig S3: The distribution of cumulative RMSD of the complex from the independent simulations. **5**

Fig S4: The distribution of molecular weight of ligands **5**

Fig S5: The distribution of binding affinities computed for 5000 protein-ligand complexes. **6**

Fig S6: Scatter plot of the Docking vs Experimental values along with the Pearson Correlation Coefficient (R_p) and Spearman rank correlation (R_s). a) Transferase b) Hydrolase c) Oxidoreductase d) Isomerase e) Ligase f) Lyase g) Others h) All. **7**

Fig S7: Scatter plot of the Calculated Binding Energy using MM-PBSA vs Experimental values along with the Pearson Correlation Coefficient (R_p) and Spearman rank correlation (R_s). a) Transferase b) Hydrolase c) Oxidoreductase d) Isomerase e) Ligase f) Lyase g) Others h) All. **8**

Fig S8: The distribution of predicted (a) Electrostatic, (b) van der Waals, (c) Non-polar Solvation free energy and (d) Polar Solvation free energy for 5000 protein-ligand complexes. **9**

N-Waters MMPBSA Calculations:

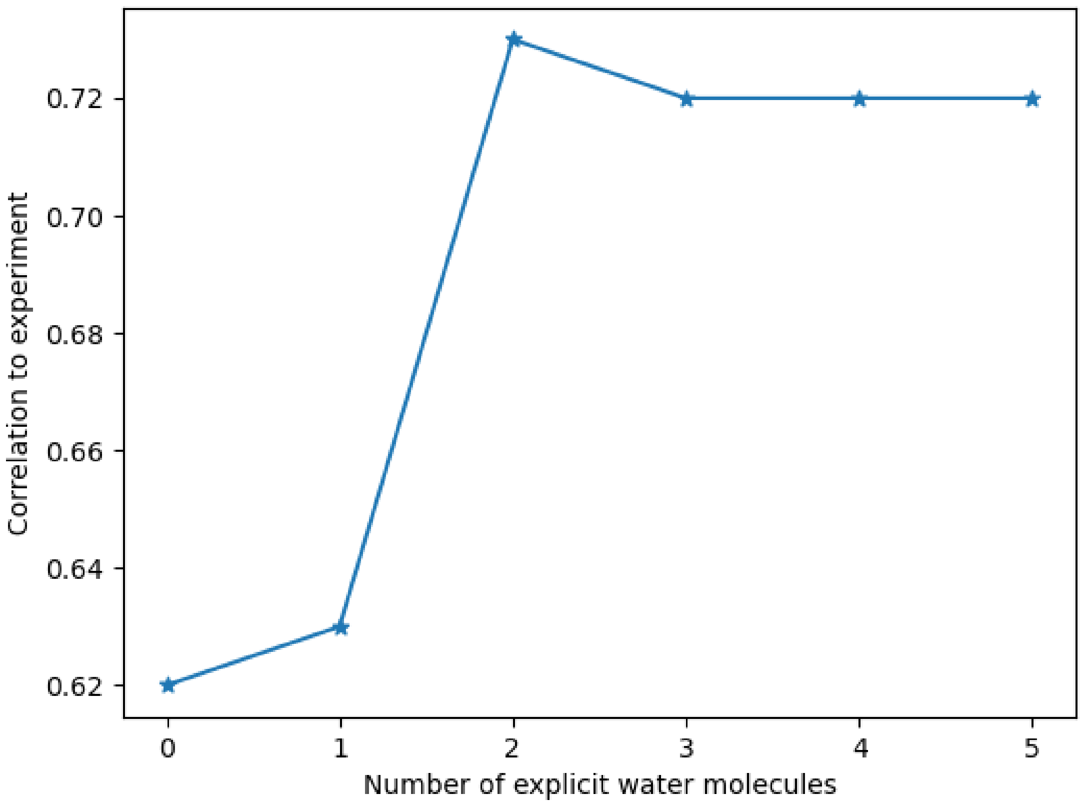

Figure S1: Change in Pearson correlation with the inclusion of explicit water molecules near protein as well as ligand at binding site in the MMPBSA calculations. This analysis performed for 75 Protein-ligand complexes which are part of 2000 PL complex set used for comparsion study.

There are reports highlighting the role of explicit water molecules in accurate prediction of binding affinities of protein-ligand complex as well as protein-protein interactions.[1–3] In order to capture possible water mediated PL interactions near the active site, we considered explicit water molecules in MMPBSA calculations and this approach is named as Nwat-MMPBSA approach. To chose the optimal number of water molecules required for accurate binding affinity prediction, we randomly selected 75 protein-ligand complexes (part of PLAS-5k dataset) whose experimental binding affinities are known. Initially binding affinities were

calculated using classical MMPBSA approach (Zero explicit water molecules). To evaluate the effect of including explicit waters we repeated the calculations on the same snapshots from MD trajectories while retaining the N closest water molecules near the active site, where N = [1, 2, 3, 4, 5]. The N waters near the active site was chosen using MDAnalysis[?] module from python. The pearson correlation coefficient between the calculated and experimentally determined affinities are plotted as a function of number of explicit water molecules and is shown in Figure S1. From Figure S1, it is clearly seen that considering two explicit water molecules gives better correlation. Hence for the remaining structures in the dataset we performed MMPBSA calculations by considering two explicit water molecules.

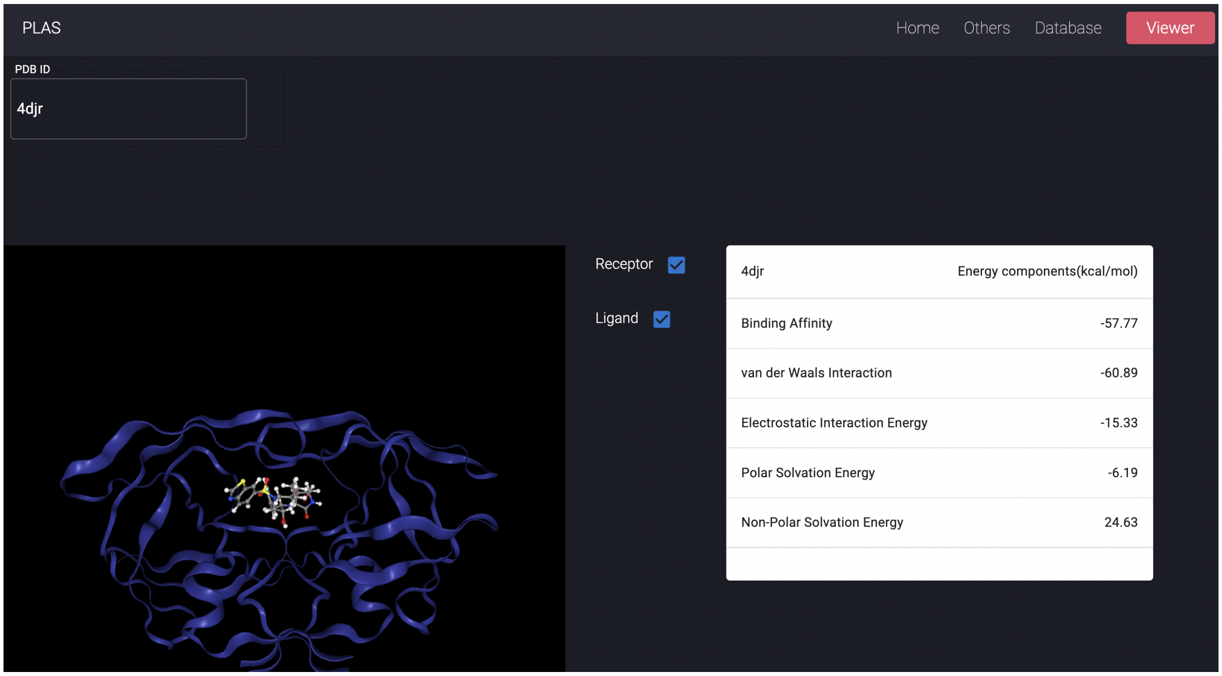

Figure S2: Screenshot of the PDB Viewer

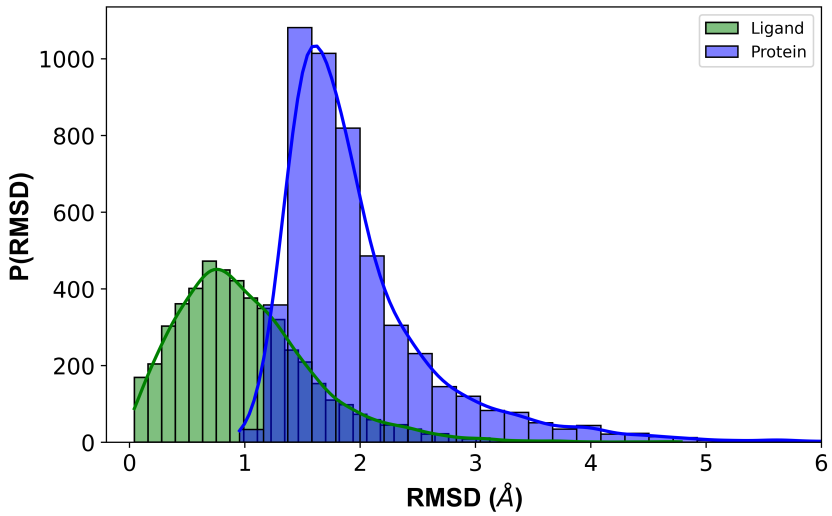

Figure S3: The distribution of cumulative RMSD of the complex from the independent simulations.

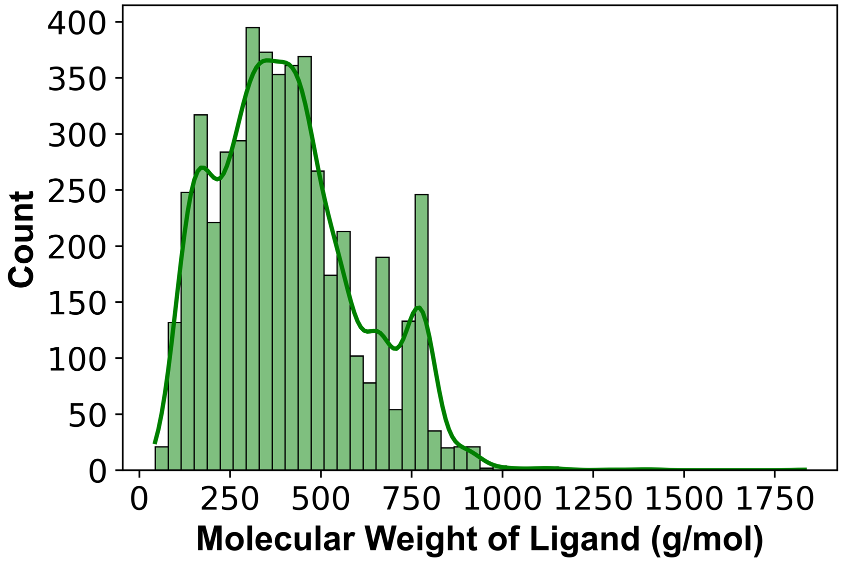

Figure S4: The distribution of molecular weight of ligands.

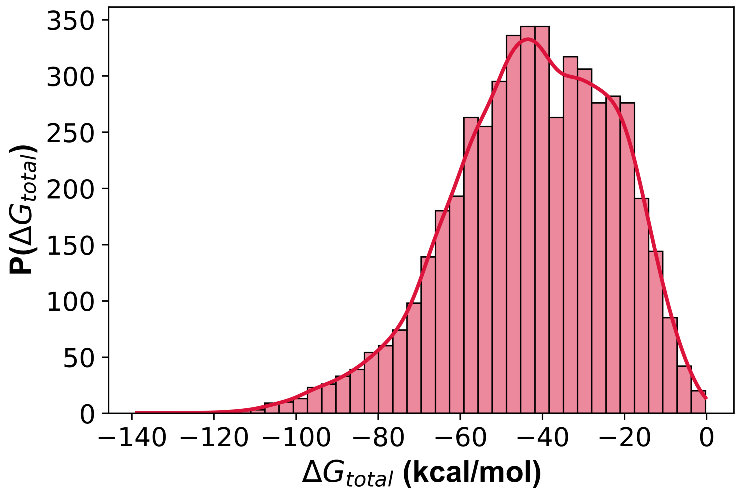

Figure S5: The distribution of binding affinities computed for 5000 protein-ligand complexes using MM-PBSA method.

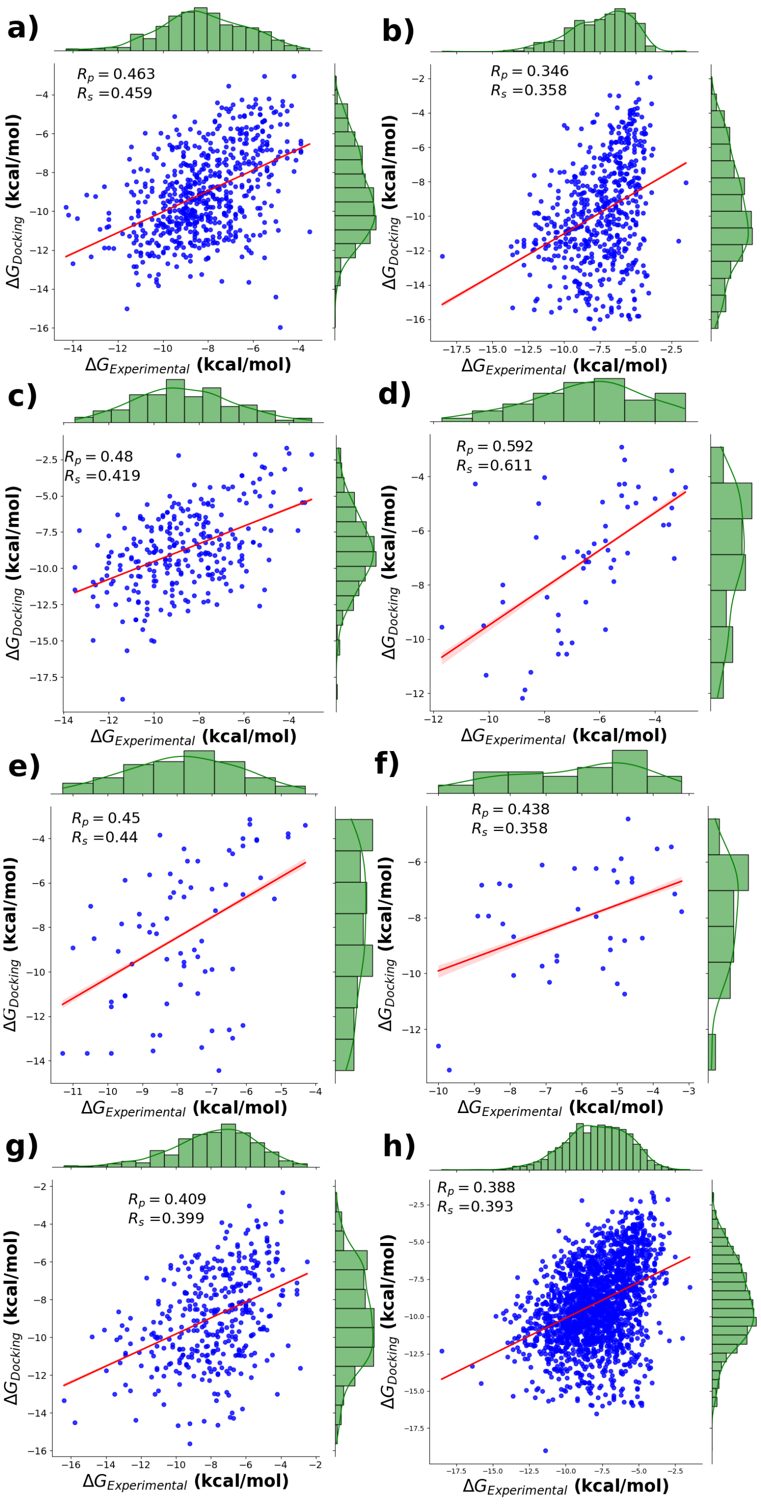

Figure S6: Scatter plot of the Docking vs Experimental values along with the Pearson Correlation Coefficient (R_p) and Spearman rank correlation (R_s). a) Transferase b) Hydrolase c) Oxidoreductase d) Isomerase e) Ligase f) Lyase g) Others h) All.

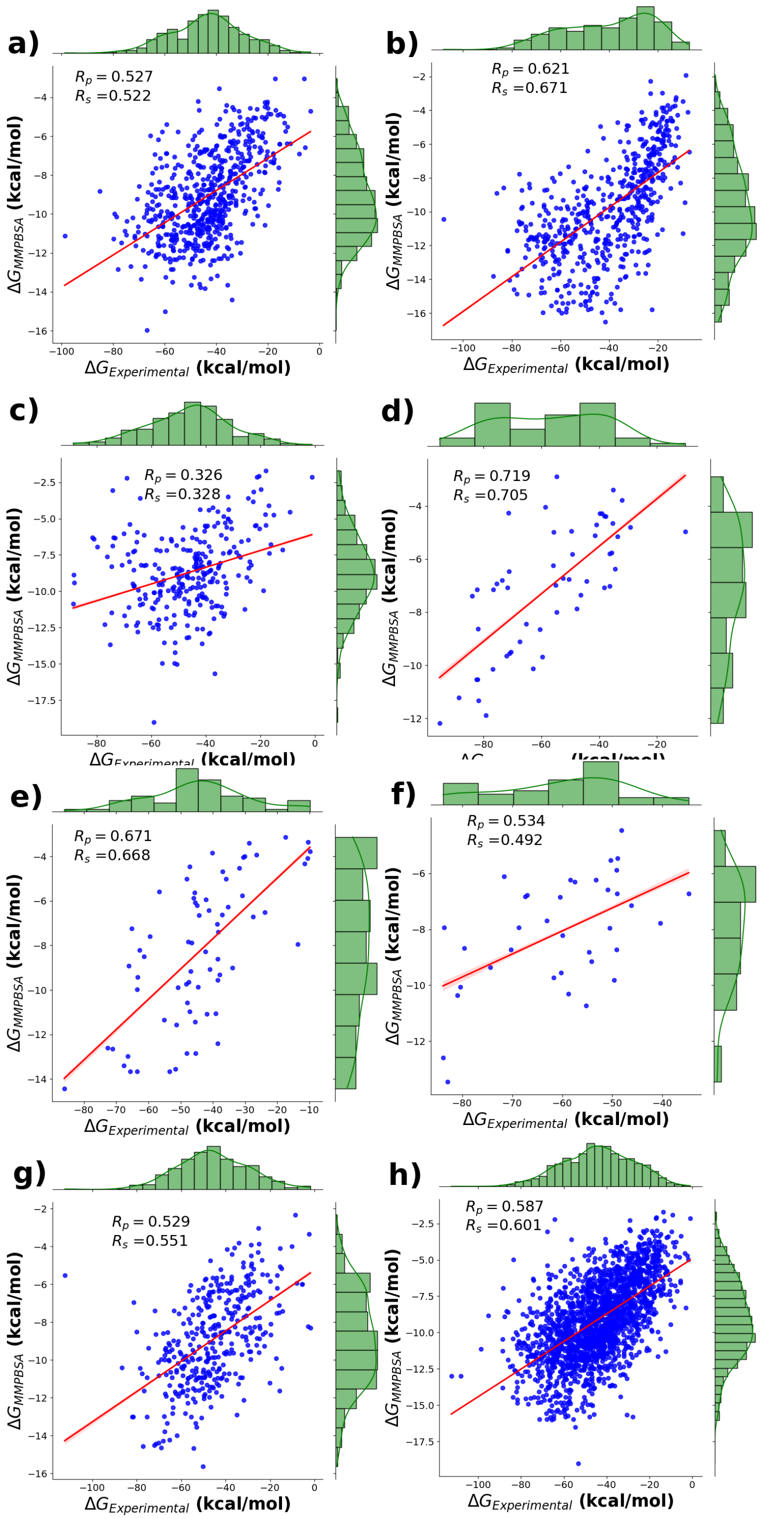

Figure S7: Scatter plot of the Calculated Binding Energy using MM-PBSA vs Experimental values along with the Pearson Correlation Coefficient (R_p) and Spearman rank correlation (R_s). a) Transferase b) Hydrolase c) Oxidoreductase d) Isomerase e) Ligase f) Lyase g) Others h) All.

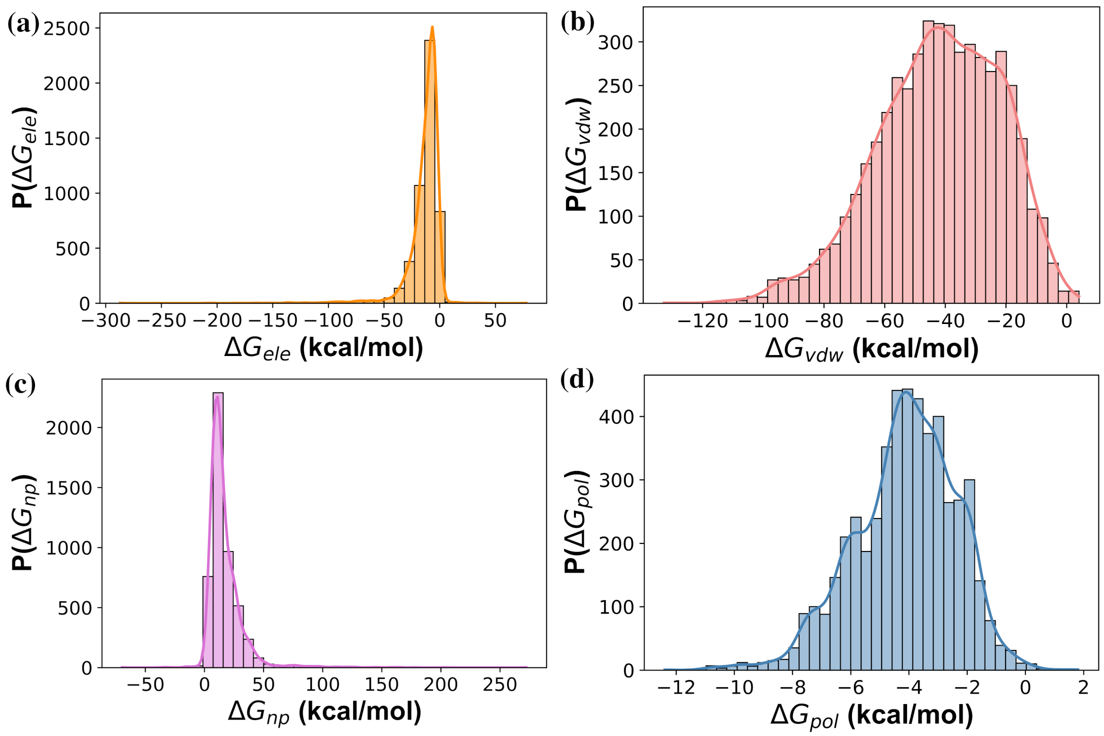

Figure S8: The distribution of predicted (a) Electrostatic, (b) van der Waals, (c) Non-polar Solvation free energy and (d) Polar Solvation free energy for 5000 protein-ligand complexes.

References

(1) Aldeghi, M.; Bodkin, M. J.; Knapp, S.; Biggin, P. C. Statistical analysis on the performance of Molecular Mechanics Poisson–Boltzmann Surface Area versus absolute binding free energy calculations: Bromodomains as a case study. *Journal of chemical information and modeling* **2017**, *57*, 2203–2221.

(2) Zhu, Y.-L.; Beroza, P.; Artis, D. R. Including explicit water molecules as part of the protein structure in MM/PBSA calculations. *Journal of Chemical Information and Modeling* **2014**, *54*, 462–469.

(3) Maffucci, I.; Hu, X.; Fumagalli, V.; Contini, A. An efficient implementation of the Nwat-MMGBSA method to rescore docking results in medium-throughput virtual screenings. *Frontiers in chemistry* **2018**, *6*, 43.
